# Acromio-Clavicular Joint Dislocation Types IV to VI: Does the Outcome with the modified Weaver-Dunn Procedure Justify the Treatment?

**DOI:** 10.5704/MOJ.1807.006

**Published:** 2018-07

**Authors:** KC Kapil-Mani, A Niroula

**Affiliations:** Department of Orthopaedics, Civil Service Hospital, Kathmandu, Nepal

**Keywords:** acromio-clavicular joint, coraco-acromial ligament, modified Weaver-Dunn Procedure, synthetic suture, tendon reconstruction procedure

## Abstract

**Introduction:** The optimal surgical treatment for acromioclavicular joint (ACJ) injuries remains controversial. The modified Weaver-Dunn (WD) procedure is one of the frequently used techniques. Recently when it was compared with anatomical autogenous tendon graft reconstruction procedures, the results were inferior. However, these anatomical procedures are technically more demanding with small margin of error and they have tendency for postoperative pain because of extra donor site incision.

**Materials and Methods:** Forty patients with type IV to VI ACJ dislocations were treated by modified WD procedure using non-absorbable synthetic suture passed through the base of coracoid process for augmentation of transferred coraco-acromial (CA) ligament. Functional outcome was assessed using the Oxford Shoulder Score, Nottingham Clavicular Score and Visual Analog Score (VAS) at the final follow-up after surgery.

**Results:** The mean pre-operative Oxford Shoulder Score improved from 25.22±2.64 (range 20 to 30) to 44.75±1.99 (range 40 to 48) and mean pre-operative Nottingham Shoulder Score improved from 49.25±4.91 (range 39 to 58) to 87.27±4.39 (range 79 to 96) at last follow-up after surgery with p-value <0.001. Thirty-five (87.5%) patients had excellent outcomes, four (10%) patients had good outcomes and one (2.5%) patient had fair outcome. Thirty-eight (95%) patients had no pain while two (5%) had moderate pain based on VAS score.

**Conclusion:** Modified Weaver-Dunn is a simple well established technique for grade IV to VI ACJ dislocation. We cannot consider this procedure as old and outdated on the argument that the long term functional outcomes are not suboptimal.

## Introduction

Acromio-clavicular joint (ACJ) disruptions account for 12% of all shoulder girdle injures in general population and 40% in athletes^1^. Most ACJ injuries (Rockwood types I to III) are managed successfully with conservative treatment except in athletes and high demand activity patients^1^. The optimal surgical treatment for ACJ injuries remains controversial. The modified Weaver-Dunn (WD) procedure is one of the most commonly used methods that involved the excision of the lateral end of clavicle and transferring the coracoacromial (CA) ligament as a substitute for ruptured coracoclavicular (CC) ligament to its lateral end. The transferred ligament was further augmented with either screw fixation, cerclage wires, autogenous tendon graft or synthetics such as dacron, carbon fibers, gore-tex and braided polyester^2,3^. Recently, the modified WD procedure was compared with synthetic ligament as well as autogenous tendon graft reconstruction revealing its inferior results in both clinical and biomechanical studies because many non-anatomical methods, even though restore the vertical stability lack in antero-posterior stability as compared to the anatomic reconstruction of CC ligaments^4-6^. Similarly Costic *et al* compared the anatomical reconstruction of semitendinosus tendon (ST) graft with the intact CC ligament showing the reconstructed ligament had the mechanical strength close to the intact ligament^7^.

There are many treatment options available for surgical reconstruction of ACJ injuries but the literature does not recommend any of these treatment options as the optimal method. Post-surgical recurrence rate of acromio-clavicular (AC) joint separation after reconstruction procedure ranges from 20 to 30% and frequently occurs within one year after initial surgery^8-10^. Preference has been given for tendon graft rather than screw fixation as an augmentation method for reconstruction procedure because of lack of implant fracture, loosening and migration of implants^11^.

However, there are certain drawbacks regarding the tendon graft reconstruction procedures, like they are technically more demanding with small margin of error, have increased postoperative morbidity because of donor site incision for tendon graft, produce suboptimal results with lack of proper preparation of tendon graft, may have risk of clavicle fracture because of large bony tunnels made for passage of graft in the clavicle and, most importantly, they lack long term significant superior results as compared to modified WD procedure^8^. We performed the modified WD procedure for ACJ injuries (type IV to VI) with use of non-absorbable synthetic suture passed through the base of coracoid process for augmentation of transferred CA ligament.

The objective of our study was to evaluate the functional outcomes of ACJ dislocations (Rockwood types IV to VI) treated by modified Weaver-Dunn procedure and to compare the results in the literature with other studies related to the reconstruction by tendon graft.

## Materials and Methods

This is a case series of prospective analytical study performed in Civil Service Hospital, Nepal, from July 2011 to June 2016. Patients with Rockwood type IV to VI acromio-clavicular joint dislocations, failed primary nonoperative treatment of type III AC joint dislocation, persistent disability and impairment for at least six months after primary treatment were included in the study. Patients with cervical spine disorders, rheumatoid arthritis, or previous surgery of the shoulder joint were excluded from this study.

**Fig. 1:**
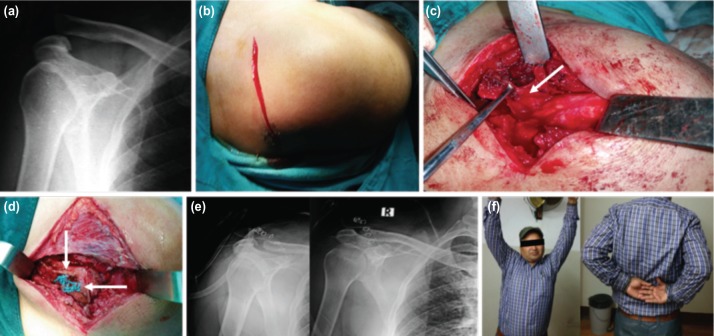
(a) Preoperative antero-posterior radiograph showing the type V acromial-clavicular joint dislocation. (b) Antero-posterior incision on lateral aspect of clavicle. (c) Resection of lateral end of clavicle and separation of coraco-acromial (CA) ligament along with bony fragment from anterior margin of acromion. (d) Two separate knots of non-absorbable Ethibond suture, medial one for the stabilisation of clavicle in reduced position by passing suture through base of coracoid process and lateral one for encroach of the bony CA ligament to lateral end of clavicle. (e) Postoperative radiograph showing the reduced clavicle at the level of acromion. (f) Motion of shoulder joint 3 months after surgery showing mild restriction on involved side.

**Fig. 2:**
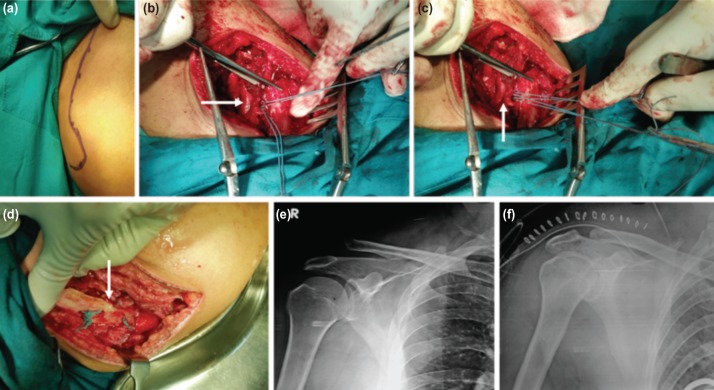
(a) Marking through the clavicle and acromian in longitudinal direction. (b,c) Resection of lateral end of clavicle and tying of clavicle in reduced position with nonabsorbable Ethibond suture passed through undersurface of coracoid process. (d) Two separate knots on lateral and medial aspect of clavicle for secure of graft and maintainance of clavicle in reduced position respectively. (e) Preoperative radiograph showing the type V AC joint dislocation. (f) Postoperative radiograph showing reduced clavicle at the level of acromion.

The procedure was performed either with regional or general anaesthesia. Folded sheets were placed behind the involved shoulder girdle to enhance the visualisation and access to the superior aspect of shoulder during surgery. A strap incision was made starting 2 to 3cm behind the AC joint and extending towards the coracoid process in an oblique fashion (Fig. 1b). Sometimes longitudinal incision was made along the length of clavicle depending upon surgeon preference (Fig. 2a). Soft tissue dissection was carried out in longitudinal direction overlying the lateral end of clavicle separating the anterior and posterior soft tissue off the bone (Fig. 1c). In acute dislocation (Fig 1a, 2e), small intra-articular disc could be visualised in the AC joint while in chronic injuries disc would be replaced by fibrous tissue. Around 1 to 1.5cm of lateral end of clavicle was resected with oscillating saw in a slightly oblique direction from supero-laterally to infero-medially to facilitate the attachment of CA ligament (Fig. 1d, 2b).

Part of the anterior fibers of deltoid muscle was released from the anterior border of acromion to visualise the CA ligament which extends from base of coracoid process to the undersurface of anterior margin of the acromion. The whole length of CA ligament was isolated from surrounding soft tissues and detached from acromion with a small piece of bone to allow bone to bone healing (Fig. 1d). Soft tissues were dissected both medially and laterally off the coracoid process and ends of two non-absorbable synthetic sutures were inserted through the undersurface of the coracoid separately with the help of right angled forceps (Fig. 2c). Two holes were prepared at 1cm interval on the superior surface of clavicle just above the coracoid process with a 3.2mm drill bit for lateral passage of the synthetic suture. At the same time a small trough was prepared at the lateral end of clavicle with a curette and two small holes were made with 2mm K wires. The clavicle was maintained in slightly over-reduced position and the two ends of one synthetic suture were passed through the previously prepared 3.2mm holes in the clavicle and tied tightly followed by tying the remaining synthetic suture (Fig. 1e, 2d). The free end of CA ligament along with bony fragment was tied with synthetic suture and passed through the two small holes made by 2mm K wires in the lateral end of clavicle. Two limbs of a suture were separately passed through the two holes and tied securely (Fig. 2d). It is important to have the ligament well seated in the lateral clavicle to facilitate healing with the cancellous bone. The anterior deltoid and posterior trapezius muscle flaps were re-approximated over the superior aspect of lateral clavicle and acromion with No.1 vicryl suture followed by closure of skin with stapler. Superior acromioclavicular (AC) ligament was found to be torn in fresh cases and often not visualised in old cases. Since lateral 1.5cm of clavicle was excised, it was not possible to repair the superior AC ligament during surgery even though it is an important contributing structure for AC joint stability.

Radiograph was done first post-operative day to view the lateral end of clavicle at the level of the acromion (Fig. 1f, 2f). A soft arm pouch sling was applied for six weeks after surgery. Passive pendulum exercise was started on Day 1 and progressed to active exercises with range of movement limited to 90 degrees of forward flexion and abduction at two weeks followed by full range of motion six weeks after surgery (Fig. 1g). A detailed pre- and post-operative clinical assessment using Oxford Shoulder Score and Nottingham Clavicle Score was performed. The questionnaires for these scores were not translated into the local language, however patients were clearly explained the meaning of each questionnaire. The former scoring system consists of 12 questionnaires related to the pain, function and disability and scores range from 0 to 48 where 0 to 19 indicates severe dysfunction, 20 to 29 moderate dysfunction, 30 to 39 mild dysfunction, and 40 to 48 satisfactory function. The latter scoring system consists of 10 questions related to pain and activities of daily living and scores range from 20 (severe dysfunction) to 100 (satisfactory function). Finally, simple Visual Analog Scale (VAS) score was used to analyse the final overall satisfaction rate which comprised straight horizontal line of fixed length with distribution of pain, as no pain (0-4 mm), mild pain (5-44 mm), moderate pain (45-74 mm), and severe pain (75-100 mm). Patients were followed up in the outpatient department for two years after surgery.

However, the final functional outcome was interpreted at the last follow-up visit (at least one year after surgery).

Statistical analysis was done by using the SPSS version 16.0. The quantitative variables were documented as mean±standard deviation. The paired t-test was used to compare the significant difference between preoperative and postoperative Oxford Shoulder Score and Nottingham Clavicle Score. A p-value of less than 0.05 was considered statistically significant.

Appropriate sample size was calculated by using the formula n = Z^2^x p x (1-p)/M^2^where n = Sample size for infinite population, Z = Z value (e.g. 1.96 for 95% confidence level), P = Prevalence or population proportion (expressed as decimal), M = Margin of Error at 5% (0.05). Since the prevalence of this injury is low, sample size in our study was considered sufficient to draw the conclusion. Distribution is normal while applying the central limit theorem in our study.

## Results

The average age of the patients in our study was 36.52±11.06 (range 22 to 74 years). All demographic data and configurations of injuries are shown in Table I. The average time interval between the injury and surgery was 19.77±13.55 (range 4 to 50 days) and average duration of operating time was 74.25±13.48 (range 40 to 97 minutes). The mean pre-operative Oxford Shoulder Score improved from 25.22±2.64 (range 20 to 30) to 44.75±1.99 (range 40 to 48) and mean pre-operative Nottingham Shoulder Score improved from 49.25±4.91 (range 39 to 58) to 87.27±4.39 (range 79 to 96) at last follow-up after surgery. Thirty-five (87.5%) patients had excellent functional outcomes, four (10%) patients had good outcome, one (2.5%) patient had fair outcome while no patient had poor outcome, based on the parameters mentioned by Jiang *et al*^12^ in Table II. Overall satisfaction with the postoperative result on the basis of a visual analog scale (VAS) was excellent. Thirty-eight (95%) patients had no pain at all, while two (5%) complained of moderate pain at final follow-up visit. Complications after this surgery were minimal as shown in Table III.

**Table I:** Demography of patients and configurations of injury

Parameters	Number (Percentage)
Gender	
Male	29 (72.5%)
Female	11 (32.5%)
Side	
Right	17 (42.5%)
Left	23 (57.5%)
Types of fractures	
Rockwood type IV	14 (35%)
Rockwood type V	24 (60%)
Rockwood type VI	2 (5%)
Duration of fractures	
Less than one month duration	27 (67.5%)
More than one month duration	13 (32.5%)
Fractures with associated injuries on other parts of body	5 (12.5%)

**Table II:** Functional outcomes of shoulder joint based on the parameters mentioned

Outcome	Pain	Motion and strength	Activity	Complete loss of reduction
Excellent	No Pain	Normal	No compromise	No
Good	Occasional ache and no analgesics needed	Normal	No compromise	No
Fair	Pain during activity requiring medication	Limited (>20 degrees difference)	Limited	No
Poor	Constant pain requiring medication	Limited(>20 degrees difference)	Limited	Yes

**Table III:** Showing the complications after modified WD procedure

Complications	Number of patients
Superficial infection	2
Ligament dislodgement	1
Stiffness of shoulder	3
Prominence of clavicle	2
Irritation of skin due to non-absorbable suture knot	2
Chronic Pain	2

## Discussion

Management of ACJ injuries remains controversial and continues to evolve over the past decade. Modalities of treatment have been changed with increasing understanding of biomechanics of the joint and nature of the problem^13^. Non-operative treatment has achieved the good results with 80 to 90% satisfaction rates for chronic grade III injuries; however, 50% of patients have residual pain and weakness. Operative treatment for acute grade III ACJ injuries has been considered over-treatment with unnecessary financial costs in cases which might have otherwise done well with conservative treatment^1^.

The average age of patients in our study was 36.52±11.06 (range 22 to 74 years), most being of the younger age. The average time interval between the injury and surgery was 19.77±13.55 (range 4 to 50 days). Twenty-seven (67.5%) AC joint dislocations were less than one month of duration while 13 (32.5%) dislocations were more than one month duration. Thus, most of the dislocations operated were of less than one month duration. There was no significant difference in the final outcomes between the old and new injuries. The mean pre-operative Oxford Shoulder Score improved from 25.22±2.64 (range 20 to 30) to 44.75±1.99 (range 40 to 48) and mean pre-operative Nottingham Shoulder Score improved from 49.25±4.91 (range 39 to 58) to 87.27±4.39 (range 79 to 96) at last follow-up after surgery with p-value <0.001. Thirty-five (87.5%) patients had excellent functional outcomes based on the parameters given by Jiang *et al*^12^ and 38 (95%) patients had no pain based on VAS score. So, these results clearly indicate that modified Weaver-Dunn procedure gives reasonably good functional outcomes at two years follow-up. It is therefore considered not an inferior or suboptimal surgical technique for grade IV to VI varieties of ACJ dislocation^14^.

Two patients had superficial infection which later healed by extended antibiotic administration and dressing at regular interval. One patient had dislodgement of graft at the lateral end of clavicle which was treated by revision surgery. Three patients had stiffness of shoulder at last follow-up. They were out of scheduled follow-up for long time and had not performed the physiotherapy on regular basis. Subsequently they were kept under physiotherapy for a prolonged duration. Two patients had prominent lateral end of clavicle and two had irritation of soft tissue because of prominent suture knot on superior surface of clavicle. Even though the complications in our study were significant in number they were considered minor and did not compromise the final functional outcome to a significant level.

A number of studies in the current literature also quoted that modified Weaver-Dunn technique was one of the most widely used surgical techniques for treatment of higher grade ACJ dislocations, with reliable functional outcomes^2,12,14^. Augmentation of transferred CA ligament could be achieved with a screw (removed later), non-absorbable heavy sutures, or suture anchors. Breslow *et al* demonstrated in a biomechanical study that similar stability can be achieved for augmentation of CC fixation with suture anchors and heavy non-absorbable sutures passed through undersurface of coracoid^15^. The suture anchor technique potentially reduced the surgical time and further risk of neurovascular injury as compared to the passage of suture through the base of coracoid process. In our study we used two non-absorbable synthetic sutures passed through the undersurface of coracoid process with the help of right angled forceps with minimal risk of neurovascular injury. Grutter and Petersen compared the three different reconstructive techniques, namely modified WD, anatomical reconstruction using flexor carpi radialis, and palmaris longus tendon graft^16^. They concluded that even though anatomical reconstruction was superior to the modified WD technique, transferred tendon graft had limited strength compared to the original ligament. Load to failure using the palmaris longus tendon graft was 326 N and that for the flexor carpi radialis tendon was 774 N. The strength in the native AC joint (815 N) was greater than any of the tendon grafts in reconstruction procedure^16^.

Concerns have been raised regarding clavicular fracture treatment for both modified WD as well as tendon reconstruction procedures. However, this problem is more frequent in tendon graft reconstruction procedures. Costic *et al* reported on two clavicular fractures in their cadaveric study. The first fracture occurred at the site of clavicle where epoxy compound was anchored and second fracture was due to inadequate bone bridge between two tunnels in the clavicle^7^. Turman *et al* reported the clavicular fracture in CC ligament reconstruction with tendon graft. It may be due to osteolysis at the site of bio-absorbable screw, relatively large size bony tunnels and subsequent cortical breach, inadequate patient compliance and lack of proper communication between surgeon and patient regarding post-operative protocol^17^. Carofino and Mazzocca indicated that clavicular fractures could be prevented by spacing the bony tunnels at least 20 to 25 mm apart^18^. Current graft augmentations, such as metal or suture cerclage techniques, have been linked to erosion of the coracoid and clavicle^19,20^.

Based on the results of our study as well as review of current literature, we can summarise that it is not mandatory to perform the tendon graft reconstruction procedures for all higher grade ACJ injuries for the following reasons. Reconstruction of ACJ using the tendon graft is a relatively longer duration surgical procedure, technically more demanding and has small margin of error that compromises the final outcomes to significant extent. It further increases the post-operative morbidity of patients because of use of extra surgical incision for donor of tendon graft. Often the lack of proper preparation of tendon graft gives the suboptimal final results. Reconstruction with tendon graft cannot reproduce the strength of dislocated joint to the level of native ACJ. Two large bony tunnels were required for passage of tendon grafts through the clavicle that rendered the bone prone to fractures more easily. More important is that the functional outcome after modified Weaver-Dunn procedure does not seem suboptimal as compared to the tendon graft reconstructive procedures except in athletes and manual laborers. The lack of control group to compare the clinical efficacy of treatment is the major limiting factor of this study.

## Conclusion

Even though different varieties of tendon graft reconstructive techniques have been reported in the literature for ACJ injuries, the modified Weaver-Dunn is a simple well established technique for grade IV to VI ACJ dislocations. We cannot consider this procedure as ineffective because many studies reported that long term functional outcomes are not suboptimal. At the same time different tendon graft reconstruction procedures have certain drawbacks, that they are technically more demanding with small margin for error, have increased postoperative morbidity because of donor site incision for tendon graft, produce suboptimal results when tendon graft preparation is not proper, and may have risk of clavicle fracture later due of large bony tunnels made for passage of graft in clavicle.

## Conflict of Interest

The authors declare that they have no conflict of interest.
